# The Final Oral/Practical State Examination at Freiburg Medical Faculty in 2012 – Analysis of grading to test quality assurance

**DOI:** 10.3205/zma000981

**Published:** 2015-10-15

**Authors:** Angela Schickler, Peter Brüstle, Silke Biller

**Affiliations:** 1 Uni Freiburg, Kompetenzzentrum Lehrevaluation in der Medizin Baden-Württemberg, Sitz Freiburg, Freiburg, Deutschland

**Keywords:** German State Examination, oral and practical part of the state examination, training of examiners, grading

## Abstract

**Aim: **The aim of this study is to analyze the grades given for the oral/practical part of the German State Examination at the Medical Faculty of Freiburg. We examined whether or not the grades given for the written and the oral/practical examinations correlated and if differences in grading between the Freiburg University Medical Center (UMC) and the other teaching hospitals could be found.

In order to improve the quality of the state examination, the medical school has been offering standardized training for examiners for several years. We evaluated whether or not trained and untrained examiners differed in their grading of the exam and how these differences have changed over time.

**Methods:** The results of the 2012 spring and fall exams were analyzed (N=315). The relevant data set was made available to us by the Baden-Württemberg Examination Office (*Landesprüfungsamt*). The data were analyzed by means of descriptive and inferential statistics.

**Results: **We observed a correlation of ρ=0.460** between the grades for the written and the oral/practical exams. The UMC and the teaching hospitals did not differ significantly in their grade distributions. Compared to untrained examiners, trained ones assigned the grade of “very good” less often. Furthermore, they displayed a significantly higher variance in the grades given (p=0.007, phi=0.165). This effect is stronger when concentrating specifically on those examiners who took part in the training less than a year before.

**Conclusion: **The results of this study suggest that the standardized training for examiners at the Medical Faculty of Freiburg is effective for quality assurance. As a consequence, more examiners should be motivated to take part in the training.

## Zusammenfassung

**Zielsetzung: **Von der Medizinischen Fakultät Freiburg werden verschiedene Maßnahmen zur Qualitätssicherung des mündlich-praktischen Teils des Zweiten Abschnitts der Ärztlichen Prüfung (M2) betrieben. Insbesondere werden die Prüfenden in einem Baden-Württemberg-weit standardisierten M2-Prüferworkshop fortgebildet.

Ziel der vorliegenden Studie war es die Notenvergabe zu analysieren. Es wurde geprüft, welcher Zusammenhang zwischen der schriftlichen und der mündlich-praktischen Notenvergabe besteht und ob sich ein Unterschied in der Notenvergabe zwischen Universitätsklinikum (UKL) und den Akademischen Lehrkrankenhäusern (ALKs) findet. Darüber hinaus wurde untersucht, ob sich die Notenvergabe von fortgebildeten und nicht fortgebildeten Prüfenden unterscheidet und inwieweit diese Unterschiede sich im zeitlichen Abstand zum Workshop verändern.

**Methodik: **Die analysierte Stichprobe (N=315) umfasst die Frühjahrs- und Herbstprüfungskohorte 2012. Der Datensatz wurde vom Landesprüfungsamt zur Verfügung gestellt und mittels deskriptiver und Inferenzstatistik ausgewertet.

**Ergebnisse: **Zwischen der schriftlichen und mündlich-praktischen M2-Prüfungsnote konnte ein Zusammenhang von ρ=0,460** ermittelt werden. Es konnte kein signifikanter Unterschied in der Verteilung der Noten zwischen UKL und den ALKs festgestellt werden. Die Daten zu den Variablen fortgebildete versus nicht fortgebildete Prüfende zeigen, dass Prüfende mit Absolvierung eines Prüferworkshops seltener die Note „sehr gut“ sowie ein breiteres Notenspektrum vergeben. In der Verteilung der Noten zeigt sich ein signifikanter Unterschied (p=0,007, phi=0,165). Dieses Ergebnis wird deutlicher bei Betrachtung von Prüfenden, deren Workshopteilnahme nicht länger als ein Jahr zurücklag.

**Schlussfolgerungen: **Die Ergebnisse der Analyse weisen darauf hin, dass der Prüferworkshop an der Medizinischen Fakultät Freiburg eine wirksame Qualitätssicherungsmaßnahme ist. Die Ergebnisse lassen den Schluss zu, die Prüfenden zur Absolvierung eines Prüferworkshops zu motivieren.

## Background

The 2002 medical licensing regulations (*Ärztliche Approbationsordnung*) [1] led to fundamental changes in the German State Examination, which then consisted of two sections until the current changes in 2012. The first section (M1) was taken by students after the first two years of study; the second one (M2) following the entire course of study, including the final practical year. Both sections are divided into a written and an oral/practical part. Some of the changes in 2002 affected the oral/practical part of the M2: its score represents one-third of the student’s final overall grade. The length of the test was extended to two full days and a practical section was explicitly introduced. A total of four to five examiners administer the examination to a maximum of four examinees [1]. It is required that the focus of this examination be on patient-oriented questions [1], [2], [3].

In general, the oral/practical M2 examination is a very extensive, professionally qualifying exam that places considerable demands on the examiners. In a 2011 resolution, the* Medizinische Fakultätentag* (MFT) made reference to the great burden placed on the medical schools. It raised concern that through this increased use of resources and the necessary expansion of the examiner pool to include people from outside the universities, a decrease in exam quality and unequal treatment of the candidates could possibly result [http://www.mft-online.de/files/200_omft_2011.pdf most recently verified on 5 Nov. 2013]. To train final-year medical students, the Medical Faculty of Freiburg cooperates with 15 teaching hospitals, which are also involved in the oral/practical M2 examinations. In order to create uniform and comparable testing practices among all of the teaching hospitals, at least one member of the university teaching staff co-administers the examinations at the teaching hospitals.

The scores assigned during the examinations should allow conclusions to be drawn about the competencies of the exam candidates. For this to be the case, grading must be based on fairness and equal opportunity, while also having the ability to stand up in court of law [4], [5], [6]. To ensure this, it is critical that a test’s strengths and weaknesses are handled with awareness, consistent testing conditions and practices are ensured, content and expectations are competently structured and standardized, and the most objective evaluation criteria possible are applied [4], [7], [8], [9]. Doing justice to the requirements for quality criteria – objectivity, reliability and validity – is a known challenge facing oral and practical examinations [6], [10], [11], [12].

An effective measure to increase the quality, and with it the quality of practical and oral exams, are training programs for examiners [4], [8], [12], [13]. During training, strategies are imparted that can optimize not only the administration of tests, but also the evaluation of test performance [6].

In Baden-Württemberg a workshop for M2 examiners was designed in 2007 by the Competency Network for Teaching in Medicine (*Kompetenznetz “Lehre in der Medizin”*) [14] and is regularly held at all the medical schools in Baden-Württemberg to prepare examiners for the oral/practical part of the exam. This workshop consists of eight instructional units divided into seminar sessions and practical exercises and pursues the objectives of creating smooth testing procedures, optimizing tests based on experience, formulating test questions and tasks, and establishing criteria-based grading [7].

In a study by Öchsner, Geiler & Huber-Lang, examiners trained in the M2 workshops conducted at the Medical Faculty of Ulm were retrospectively surveyed about the effects and sustainability of the workshop using self-evaluation questionnaires [7]. The examiners were asked about the core issues of responding to strengths and weaknesses in the M2 exam with awareness, knowledge regarding the influence of reliability and validity on the oral/practical exam, confidence in designing test questions and following formal rules and regulations, and implementing a structured oral examination. The responses to all of the core issues pointed to benefits resulting from the examiner workshop, and these could be detected in the examiners even two years after completing the workshop. In close relation to the examiner perspectives presented in this study, our analysis here focuses on the output – the assignment of grades – at the Medical Faculty of Freiburg.

## Aim

The aim of this study is to analyze the grading of the oral/practical M2 examination at the medical school in Freiburg in order to assess the internal quality assurance measures. The following four aspects were scrutinized:

- The correlation between written and oral/practical M2 grades;
- The difference in grading at the hospitals where testing was conducted: University Medical Center (UMC) versus the teaching hospitals;
- The difference in scoring between the examiners with and without prior training in one of the workshops;
- In-depth analysis of workshop sustainability in regard to grading.

## Method

To analyze the assignment of grades for the oral/practical M2 exams, the Examination Office of Baden-Württemberg (*Landesprüfungsamt)* provided the anonymized data for the examinees in the 2012 spring and fall cohorts. Information was available on the candidates’ written and oral/practical scores, as well as on the corresponding examination committees. In addition, it was known which examiners had previously completed the training workshop.

The size of the sample of examinees was N=315. The data set encompassed a total of 94 examination committees. An exam candidate was assigned to trained examiners for the analysis if one committee member had undergone training. The data were then entered and processed in SPSS (2012, version 20). Analysis was performed on the basis of the conventions of Bühner & Ziegler [15] and Bortz [16] and included descriptive statistical calculations and theory-based testing of hypotheses using inferential statistics.

## Results

First, the correlation between the M2 examinees’ scores on the written part and the oral/practical part was analyzed. The written scores displayed a mean value (M) of 2.45 (standard deviation (SD)=0.744). In contrast, a higher mean was calculated for the scores for the oral/practical part (M=1.92, SD=0.716). The grade of “very good” was assigned much more frequently for performance on the oral/practical part than for written test performance. Between the written and oral/practical scores, a slight linear correlation was seen with a highly significant rank correlation coefficient of ρ=0.460**.

It was then investigated if differences existed between the assignment of grades at the UMC and the other teaching hospitals. The mean and standard deviation for the UMC were M=1.95 and SD=0.768; for the teaching hospital these were M=1.88 and SD=0.661. Applying the Mann-Whitney U test, no significant difference between the two hospital groups was detected (ρ=0.682, Φ=0.023).

In addition, we checked for differences in the grading depending on if the oral/practical M2 exam was administered by examiners who had previously attended the workshop. The dichotomous variable was defined in that we evaluated whether or not an individual candidate was examined by a committee composed of examiners who had or had not undergone specific training. The analysis of the data for those with workshop training showed a mean of M=1.99 (SD=0.741); for those without the workshop the value is M=1.70 (SD=0.634). Using the Mann-Whitney U test, it could be shown that a highly significant difference of ρ=0.007**, with a weak effect of F=0.165, exists between the two groups regarding the distribution of the grades.

This calculation was carried out again in regard to the sustainability of the M2 examiner workshop. Here, the variable regarding the length of time between administering the exam and attending the workshop was evaluated. Examination committees were considered trained if workshop attendance had taken place no less than one year ago. For this group, the trained examiners displayed a mean of M=2.08 (SD=0.782). For the group of examiners who had not received training (or had attended the workshop over a year ago), the following value was determined: M=1.72 (SD=0.642). With the Mann-Whitney U test, a highly significant difference of ρ=0.000** was calculated with a small effect Φ=0.233.

A summary of these results is presented in Table 1 (Tab. 1) and Table 2 (Tab. 2).

## Discussion and Conclusions

The analysis of the grading was able to show that the efforts put into quality assurance at the Medical Faculty of Freiburg are worthwhile.

A highly significant correlation of ρ=0.460** was determined between the written and oral/practical grades. When focusing on the descriptive data analysis, it is seen that the scores assigned for oral/practical performance are on average better than those given for the written part. The grade of “very good” is assigned much more frequently for oral/practical performance than for written. This phenomenon has also been described in the literature [17].

The results of the other investigated aspects reveal no meaningful differences between the UMC and the other teaching hospitals concerning grading for the oral/practical M2 exam. This result can be viewed as an indication that the efforts to establish a uniform and comparable testing practice at all the hospitals are effective and should be retained at the Medical Faculty of Freiburg.

The results regarding the difference between candidates who were examined by committees with trained members or those without show a highly significant difference of p=0.007** with a small effect Φ=0.165. When looking carefully at the descriptive data, it is seen that committees with members who have attended the workshop give the grade of “very good” more seldom and assign a wider range of grades overall.

When examining the sustainability of the workshop (committees were defined as trained if the workshop had taken place less than one year prior), the result is even clearer: p=0.000** with the effect of Φ=0.233. The results presented here contrast with the study done by Öchsner, Geiler & Huber-Lang, in which evidence was found showing workshop sustainability over a period of two years on the basis of self-evaluation by the examiners [7]. Pertinent effects should be investigated in more detail in subsequent studies. Moreover, the role of the examination committee chairman should be included in the analysis. In the present study it was not possible to examine this aspect more closely.

Whether or not the observed change in examiners’ grading after completing the workshop is accompanied by a higher quality of testing cannot be conclusively established. A positive effect of the workshop on grading does appear to be present, since the content imparted during the workshops is meant to assist in adequately assigning grades in terms of quality criteria.

In conclusion, it can be asserted that the M2 workshop helps achieve the highest quality possible for the oral/practical M2 examinations administered at the Medical Faculty of Freiburg. As a result, the M2 workshop can be recommended for all examiners [4], [7], [8], [9].

However, it must be pointed out that subject-specific testing cultures demonstrate a high level of stability over time when it comes to determining grades [4]. For this reason, it is recommended that any existing quality assurance measures regarding test quality be retained and intensified in order to effect long-term change in the testing practices related to grading. An indication for this could also be the higher significance for the analysis of the sustainability and the slight increase in effect size. In respect to this, a regular review of the knowledge gained in the M2 workshop should be considered; however, further studies are needed on the sustainability of acquired grading practices and of testing cultures in general.

## Competing interests

The authors declare that they have no competing interests.

## Figures and Tables

**Table 1:**
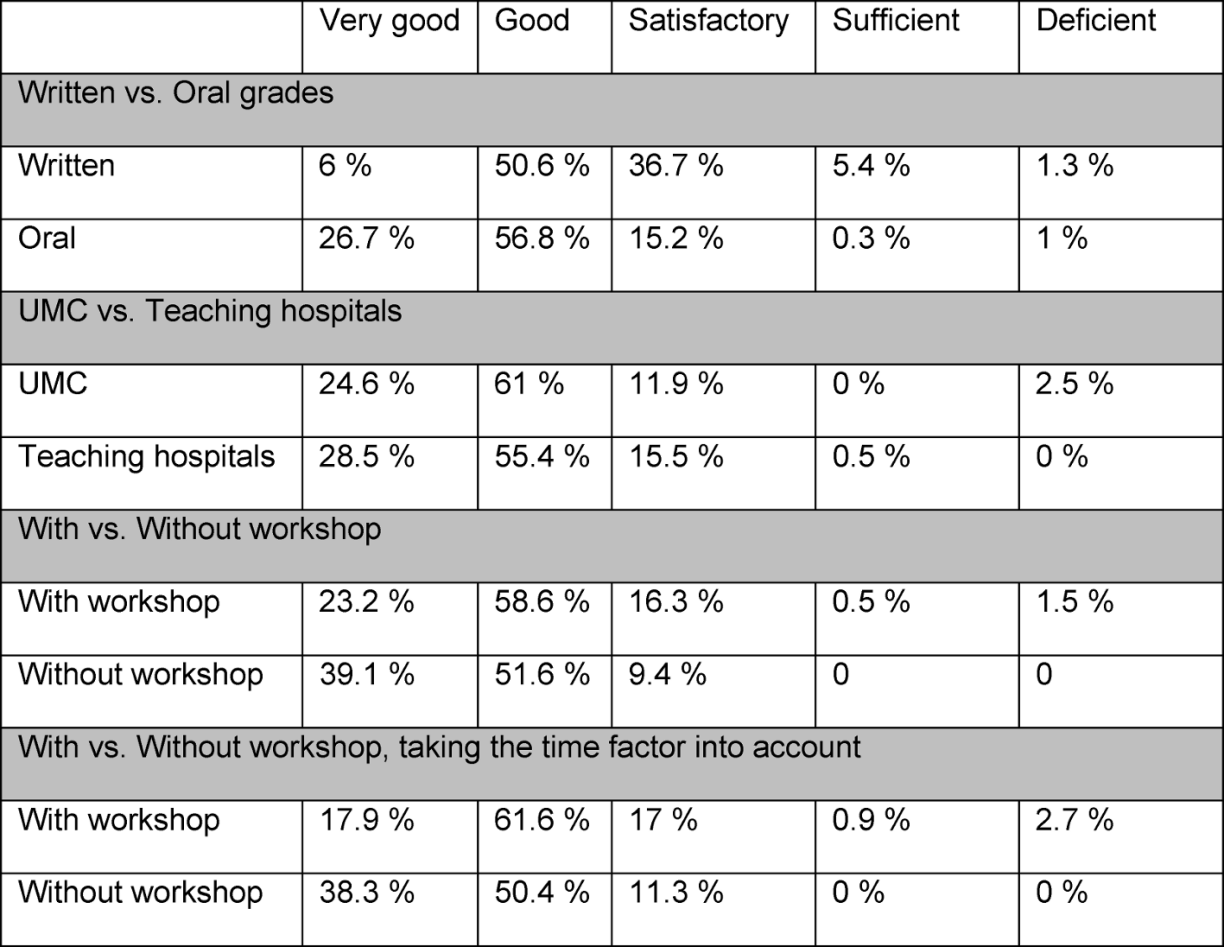
Distribution by percentage

**Table 2:**
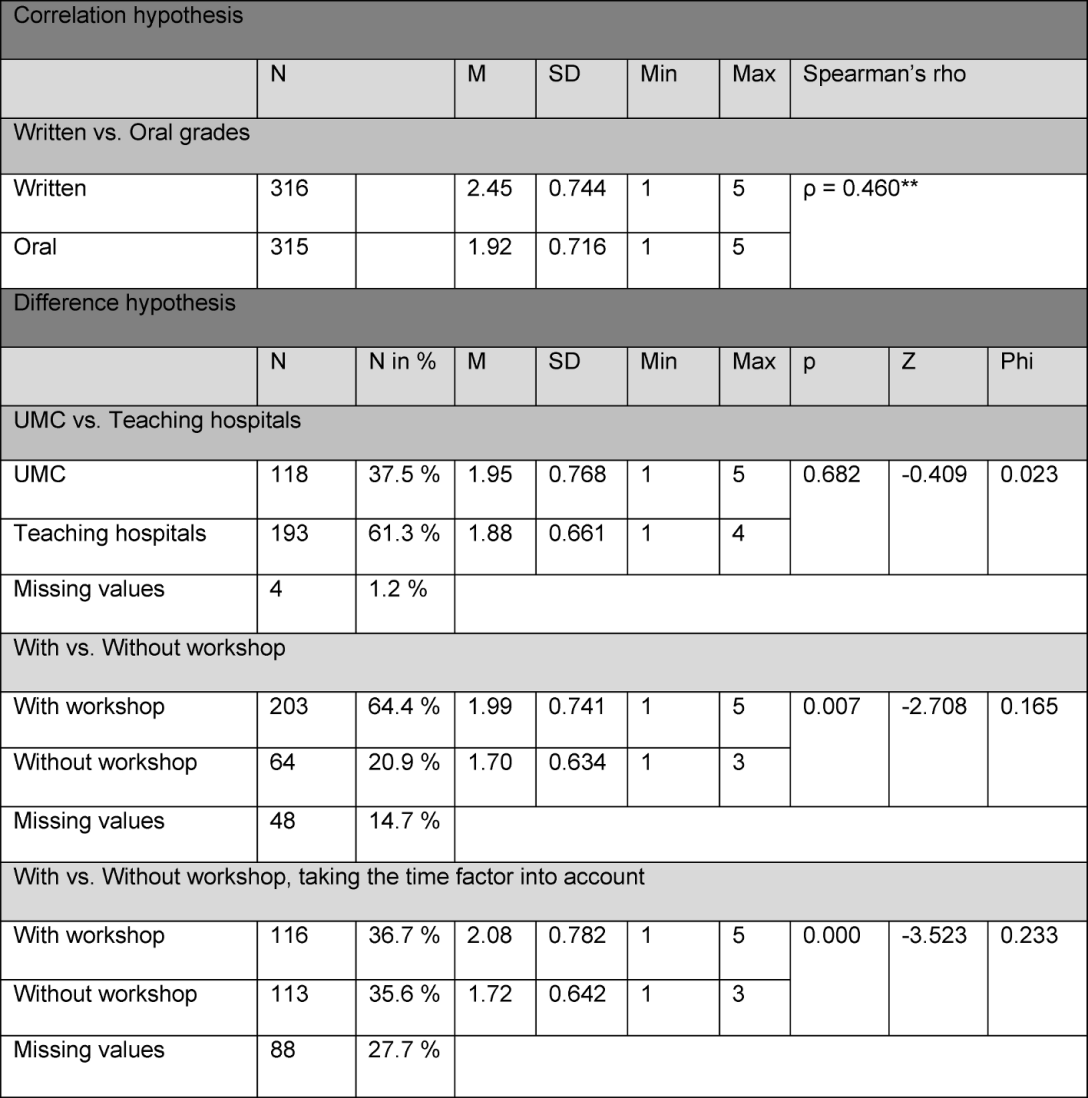
Descriptive statistics
